# The Complex Relationship between Metals and Carbonic Anhydrase: New Insights and Perspectives

**DOI:** 10.3390/ijms17010127

**Published:** 2016-01-19

**Authors:** Maria Giulia Lionetto, Roberto Caricato, Maria Elena Giordano, Trifone Schettino

**Affiliations:** Department of Biological and Environmental Science and Technologies (DiSTeBA), University of Salento, Via Prov.le Lecce-Monteroni, 73100 Lecce, Italy; roberto.caricato@unisalento.it (R.C.); elena.giordano@unisalento.it (M.E.G.); trifone.schettino@unisalento.it (T.S.)

**Keywords:** carbonic anhydrase, metals, inhibition, expression, biomarker, bioassay

## Abstract

Carbonic anhydrase is a ubiquitous metalloenzyme, which catalyzes the reversible hydration of CO_2_ to HCO_3_^−^ and H^+^. Metals play a key role in the bioactivity of this metalloenzyme, although their relationships with CA have not been completely clarified to date. The aim of this review is to explore the complexity and multi-aspect nature of these relationships, since metals can be cofactors of CA, but also inhibitors of CA activity and modulators of CA expression. Moreover, this work analyzes new insights and perspectives that allow translating new advances in basic science on the interaction between CA and metals to applications in several fields of research, ranging from biotechnology to environmental sciences.

## 1. Introduction

Carbonic anhydrase (CA) is a widely-distributed metalloenzyme, which catalyzes the reversible hydration of CO_2_ to HCO_3_^−^ and H^+^. This biochemical reaction plays a key physiological role in diverse biological systems. Six distinct and unrelated CA families (α-, β-, γ-CA, δ, ζ and η-CAs) have been identified in animals, plants, algae and bacteria [1,2]. They all catalyze the same reaction of CO_2_ hydration, but each family shows proper specific characteristics in primary amino acid sequence and 3D tertiary structure.

In animals, CA isoforms play a fundamental role in a number of physiological processes involving carbon dioxide and bicarbonate, such as transport of CO_2_ and HCO_3_^−^ between body tissues and respiratory surfaces, pH homeostasis, electrolyte transport in various epithelia, biosynthetic reactions (gluconeogenesis, lipogenesis and ureagenesis), bone resorption and calcification. In algae, plants and some bacteria, CA isoforms are fundamental for photosynthesis [3,4].

The α-carbonic anhydrases are monomeric or dimeric and are found in animals, some fungi, bacteria, algae and green plants [5]. In mammals, at least 16 different α CA isoforms were isolated. Mammalian CA isoforms CAI, II, III, IV, VA, VB, VI, VII, IX, XII, XIII, XIV and CA XV (not expressed in humans) have catalytic activity, while the remaining three CAs (CARP VIII, CARP X and CARP XI) have lost the catalytic activity and are known as CA-related proteins [6].

The β-carbonic anhydrases are dimers, tetramers or octamers and are expressed mainly in fungi, bacteria, archaea, algae and chloroplasts of monocotyledons and dicotyledons [7] and some prokaryotes [8].

The γ-anhydrase class is a homotrimer that has been described in bacteria, Archaea and plants [9]. It also includes a number of non-catalytically-active homologs present in diverse species. δ- and ζ-CAs are present in several classes of marine phytoplankton. The δ class has been described in diatoms, and its prototype is the CA TWCA1 from the marine diatom *Thalassiosira weissflogii* [10,11]. The ζ-CAs are probably monomers and have three slightly different active sites on the same protein molecule [12].

The η-CA was recently found in a number of species of the *Plasmodium* genus. These are a group of enzymes previously ascribed to the α family, but recently demonstrated to have a number of unique features, including their metal ion coordination pattern [2].

This review focuses on an interesting aspect of the research on CA, the relationships between carbonic anhydrase and metals, which play a fundamental role in the bioactivity of this metalloenzyme. The review points out the complexity and multi-aspect nature of these relationships, since metals can be cofactors of CA, but also inhibitors of CA activity and modulators of CA expression. New insights and perspectives are discussed encompassing several fields of research from biotechnological applications to environmental sciences.

## 2. Metals and CA Catalytic Site

**Figure 1:**
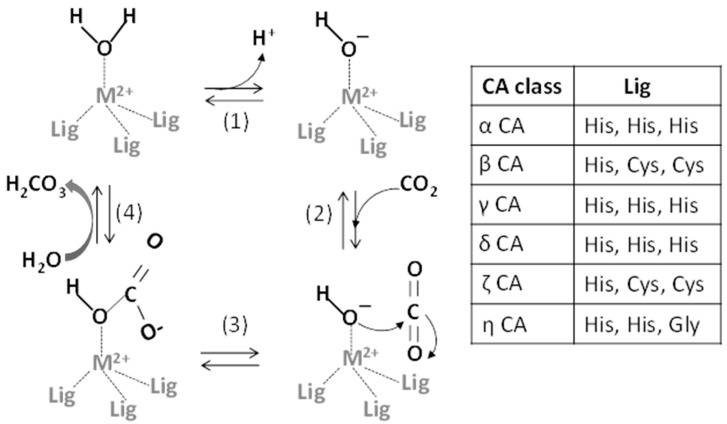
The reversible hydration of carbon dioxide to bicarbonate catalyzed by CAs by means of a metal (M)-hydroxide mechanism. Modified from Berg [17]. (**1**) The release of a proton from the zinc-bound water generates the zinc-bound OH^−^; (**2**) A CO_2_ molecule binds to the active site and is positioned for optimal interaction with the zinc-bound OH^−^; (**3**) The hydroxide ion attacks the carbonyl of CO_2_, producing HCO_3_^−^; (**4**) The release of HCO_3_^−^ regenerates the enzyme.

All CA isoenzymes catalyze the reversible hydration of CO_2_ to HCO_3_ and H^+^ through a metal-hydroxide [Lig^3^M^2+^(OH)^−^] mechanism [13,14,15] (Figure 1). The central catalytic step involves the reaction between CO_2_ and the OH^−^ bound to the zinc ion, yielding a coordinated HCO_3_^−^ ion, which is subsequently displaced from the metal by H_2_O. In the α-, γ- and δ-CA classes, Lig^3^ is represented by three key amino acid residues, which are three histidines in α-CA, γ-CA and δ-CA, one histidine and two cysteines in β-CA and ζ-CA and two His and one Gly residues in η-CA [16]. A fourth histidine, that is His 64 in human CAII (the most investigated CA isoform), not directly part of the active site, contributes to the catalytic process representing the so-called “proton shuttle”. This allows the H^+^ transfer from the metal-bound water molecule to buffer molecules located outside the active site and ensures the reaction of the metal-bound OH^−^ with CO_2_ to produce HCO_3_^−^.

The metal (M) in the carbonic anhydrase metal-hydroxide [Lig^3^M^2+^(OH)^−^] mechanism is Zn^2+^ for all classes, but other transition metals have been demonstrated to bind to the catalytic site as physiologically-relevant metal cofactors or displacers of the native cofactor, producing in this case new CA metallovariants (Table 1).

**Table 1:** Metals as physiologically-relevant cofactors of CA.

CA Families	Metals as Physiologically-Relevant CA Cofactors	Ref.
α-CA	Zn^2+^	[21]
β-CA	Zn^2+^	[7]
γ-CA	Fe^2+^; Zn^2+^	[9,22]
δ-CA	Zn^2+^	[10]
ζ-CA	Cd^2+^; Zn^2+^	[12]
η-CA	Zn^2+^	[2]

Apart from zinc, other metals have been found to be physiologically-relevant cofactors of some CAs.

### 2.1. Metals as Physiologically-Relevant Cofactors of CA

**Figure 2:**
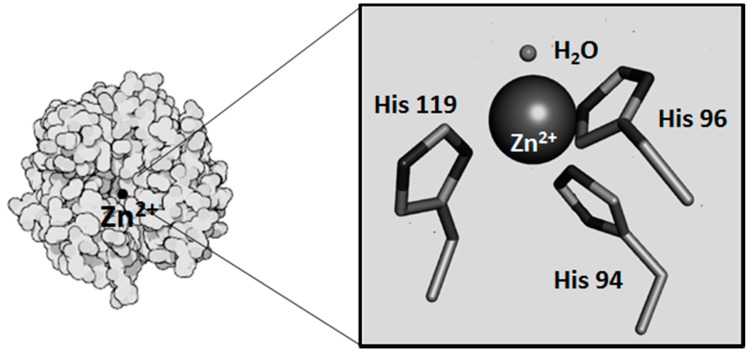
Human CAII: in detail, the metal binding site with the zinc ion as a sphere, the direct ligand histidines, H94, H96, H119, and the water molecule. Modified from Mahon *et al* [31] and from Dutta and Goodsell [32].

Zn^2+^ is one of the most widely-used metallic elements as enzyme cofactor in nature, and its presence in all of the CA families is a successful confirmation of its peculiar properties. The reason for its success lies in the filled *d* orbital (d10). Unlike other first-row transition elements (e.g., Sc^2+^, Ti^2+^, V^2+^, Cr^2+^, Mn^2+^, Fe^2+^, Co^2+^, Ni^2+^ and Cu^2+^), Zn^2+^ is not involved in redox reactions, but rather, it acts as a Lewis acid accepting a pair of electrons [18]. This makes zinc a good metal cofactor for biochemical reactions requiring a redox-stable ion to function as a Lewis acid-type catalyst [19], such as proteolysis and carbon dioxide hydration. Zinc complexes have low thermodynamic stabilities, as well as variable geometries, which in turn account for low activation barriers. This makes zinc a versatile and suitable as an active site metal [20]. Zinc is in the +2 state, and it is positioned in a cleft in the center of the CA molecule (Figure 2). It is coordinated by the three key amino acid residues (see above). The fourth coordination site is occupied by a water molecule. The role of Zn^2+^ in the CA catalytic mechanism is to promote the deprotonation of H_2_O with the production of the nucleophilic OH^−^, which in turn can attack the carbonyl group of CO_2_ to convert it into HCO_3_^−^. A water molecule subsequently displaces the bicarbonate at the metal (Figure 1).

Fe^2+^ has been demonstrated to be a physiological metal cofactor of γ-CAs [22,23]. The γ-CA class is among the most ancient, with homologs widespread in Archaea and Bacteria [24]. CAM, the prototypic γ-class CA from the anaerobic Archaea species *Methanosarcina thermophila*, has been demonstrated to contain zinc in the active site when overproduced in *Escherichia coli* and purified in aerobic experimental conditions [22]. On the other hand, when the enzyme is purified anaerobically, it has been demonstrated to contain Fe^2+^ in the active site (Fe-CAM) and to have a three-fold increased activity. These contrasting results are explained by the fact that in aerobic conditions, Fe^3+^ is oxidized and is rapidly substituted by Zn^2+^ present in the reaction buffers not treated with chelating agents. The stability of complexed Zn^2+^ is much greater than that for Fe^2+^ ligated with the nitrogen atoms of histidine residues coordinating the active-site metal in CAM. Thus, aerobic purification results in the loss of Fe^3+^ and substitution with Zn^2+^. These findings clearly suggest Fe^2+^ as the physiologically-relevant metal [22,23,24] in the active site of the CAM enzyme. Considering that Fe^2+^ is available in oxygen-free environments, the finding of iron as a physiological metal cofactor in CAM from an anaerobic species suggests the possibility that iron could act as a metal cofactor in γ-class CA enzymes and possibly in other classes in anaerobic organisms [23]. Moreover, evidence for the role of Fe^2+^ in CA catalytic activity has been found also in the α-class. In fact, in duck erythrocytes, the addition of Fe^2+^ in the reaction medium increases CA activity [25].

Cd^2+^ has been demonstrated to be naturally used as a catalytic metal in the cadmium-carbonic anhydrase (CDCA1), a ζ-CA isolated from the marine diatom *Thalassiosira weissflogii* [26,27,28]. Its homolog gene has been found in a number of diatom species, as well as natural assemblages [12]. CDCA1 provides the first evidence of the biological role of cadmium, which is usually considered a toxic element associated with environmental pollution [29]. CDCA1 is considered a “cambialistic” enzyme because it can use either Zn or Cd for the catalytic reaction [12]. The ability to use Cd in CDCA allows the diatom *T. weissflogii* to support the needs of fast growth also in Zn^2+^ limiting conditions, and this has ecologically relevant implications. In fact, in the oceans, which are known to be poor in metals, this ability might have given a competitive advantage to diatoms with respect to other species, contributing to the radiation of diatoms during the Cenozoic Era and to the parallel decrease in atmospheric CO_2_ [12]. In the ocean, Cd shows a nutrient-like concentration profile, with a low concentration at the surface, because of phytoplankton uptake, and an increased concentration at depth due to remineralization of sinking organic matter. This Cd profile is thought to be explained by the use of this metal by CDCA.

Co^2+^ has been demonstrated to *in vivo* substitute Zn^2+^ in the other genetically-distinct CA form (TWCA1) (δ-CA) isolated from the diatom *T. weissflogii* [30]. Although the affinity of TWCA1 for Co^2+^ is lower than for Zn^2+^ and the Co-substituted enzyme is less active than the Zn-form [30], it has been suggested that the Co^2+^ substitution can alleviate the Zn^2+^ limitation in diatoms in open oceans, as also suggested for Cd^2+^ [26,30].

### 2.2. Metals as Displacers of the Native Metal Cofactor

In addition to the role of some metals as CA physiologically-relevant metal cofactors, many divalent metal ions, such as Co^2+^, Ni^2+^, Mn^2+^, Cu^2+^, Cd^2+^ and Hg^2+^, have been demonstrated to easily bind to the three-histidine moiety within the CA active site in *in vitro* experimental conditions [33,34,35]. Most of this information comes from transmetallation experiments on the α-CAII. The CO_2_ hydration catalytic activity of metal-substituted human CAIIs, relative to wild-type CAII activity, is about 7% for the Mn^2+^-substituted CA, about 2% for the Cd^2+^-substituted and about 0% for the Cu^2+^-substituted and Hg^2+^-substituted ones, respectively [36,37]. Only Co^2+^ is able to produce a catalytically-functioning enzyme with a wild-type catalytic efficiency (*K*_cat_/*K*_m_ = 8.7 × 10^7^ M^−1^·s^−1^ for Zn^2+^ *vs.* 8.8 × 10^7^ M^−1^·s^−1^ for Co^2+^) [34] because only cobalt besides zinc has a tetrahedral coordination at a pH around eight. In particular, Co-CA represents an ideal metallovariant for studying the CA catalytic site. In contrast to Zn^2+^, lacking a spectroscopic signature, the paramagnetic Co^2+^ is accessible to spectroscopic analysis, providing information about the environment of the metal ions in the CA active site [38].

### 2.3. Metal Binding to Other Sites in the CA Protein

In addition to the ability of some metals to bind the three key amino acid residues of the CA active site, metals are also able to bind CA protein in other sites elsewhere in the molecule thanks to their affinity for thiol and histidyl groups. Mercuric ions have been demonstrated by X-ray crystallography [39] to bind to the His-64 ring (the so-called proton shuttle) and also to Cys-206 of CAII [40]. Copper ion is known to bind to the imidazole of His-64 in CAII, preventing its function in the H^+^ transfer from the zinc-bound H_2_O to buffer molecules outside the active site [41]. In addition, Cu^2+^ is also able to bind CA in another binding site [42] nearby the N-terminus of the proteins, presumably in a four-coordinate tetragonal Cu^2+^ site with two histidine ligands and an N-terminal amine [43]. Because of the presence of this high affinity site, CA seems to be a member of a growing number of proteins and peptides that have been found to have an N-terminal Cu^2+^-binding site, including serum albumin and amyloid β-peptide complexes [43]. It is unknown why CA has this N-terminal Cu^2+^ site that does not show any catalytic activity. It is possible that this site could function as a sequestering site of adventitious Cu^2+^. In this respect, an intriguing hypothesis suggests that this N-terminal site can be effective in defending the native zinc(II)-(His)3 active site of CA, as well as other metalloproteins, from Cu^2+^ displacement, and in turn, inactivation [43].

## 3. Metals and CA Activity Inhibition

The ability of some transition metals either to displace Zn^2+^ in the active site or to bind histidine and cysteine residues in sites other than the active site accounts for the inhibitory effect of some metals on CA activity, reported by several authors in a number of tissues in a variety of animal species. Table 2 summarizes the results obtained in *in vitro* studies.

In the teleost fish *Ictalurus punctatus*, Cd^2+^, Cu^2+^, Ag^+^ and Zn^2+^ were demonstrated to inhibit erythrocyte CA activity [44]. In the euryhaline teleost *Anguilla anguilla*, a significant *in vitro* tissue-specific inhibition of branchial and intestinal CA activity by Cd^2+^ was found [45,46]. In particular, in the intestine, a cytosolic and a membrane-bound CA were detected, with the cytosolic isoform more sensitive to the metal than the membrane-bound one. In the European seabass (*Dicentrarchus labrax*), Al^+3^, Cu^+2^, Pb^+2^, Co^+3^, Ag^+1^, Zn^+2^ and Hg^+2^ were found to be competitive inhibitors of liver CA activity [47]. In the liver of sea bream (*Sparus aurata*) Ag^+^, Ni^2+^, Cd^2+^ and Cu^2+^ were shown to be noncompetitive inhibitors of CA activity [48].

In rainbow trout (*Oncorhynchus mykiss*), Co^2+^, Cu^2+^, Zn^2+^, Ag^+^ and Cd^2+^ inhibited brain CA activity. Ag^+^ and Cd^2+^ were competitive inhibitors, Cu^2+^ was noncompetitive inhibitor, while Zn^2+^ was uncompetitive inhibitor [49].

In the estuarine crab *Chasmagnathus granulate*, Cd^2+^, Cu^2+^ and Zn^2+^ inhibited branchial CA activity both *in vitro* and *in vivo* [50]. In other euryhaline crabs, *Callinectes sapidus* and *Carcinus maenas*, a significant *in vitro* inhibition of gill CA by Ag^+^, Cd^2+^, Cu^2+^ and Zn^2+^ was demonstrated, with strong differences between the gill CA of the two species, *Callinectes sapidus* CA being more sensitive than *Carcinus maenas* CA [51]. In humans, the cytosolic HCAI and II were *in vitro* inhibited by Pb^2+^, Co^2+^ and Hg^2+^. Pb^2+^ was noncompetitive inhibitor for HCAI and competitive for HCAII, Co^2+^ was competitive for HCAI and noncompetitive for HCAII, and Hg^2+^ was uncompetitive for both isoforms [52].

In the sturgeon *Acipenser gueldenstaedti*, Ag^+^, Zn^2+^, Cu^2+^ and Co^2+^ were weak inhibitors of the erythrocyte CA activity [53]. In sheep kidney, Pb^2+^, Co^2+^, Hg^2+^, Cd^2+^, Zn^2+^, Se^2+^, Cu^2+^ and Al^3+^ competitively inhibited CA activity in the low molar/millimolar range [54]. In the Turkish native chicken, Al^3+^, Hg^2+^, Cu^2+^, Pb^2+^ and Cd^2+^ were competitive inhibitors of erythrocyte CA activity. Pb^2+^ exhibited the strongest inhibitory action; Cd^2+^ and Hg^2+^ were moderate inhibitors, while Al^3+^ and Cu^2+^ were weaker inhibitors [55].

Ki or IC_50_ values for *in vitro* metal inhibition of CA activity range from micromolar to millimolar depending on species, tissue and isoform. Although in some cases the Ki or IC_50_ values are in the submillimolar or millimolar range, suggesting a weak inhibition, they may be significant for infield inhibition due to the bioaccumulation process occurring for most metals in many exposed organisms under prolonged exposure [56].

**Table 2:** *In vitro* inhibition of CA activity by trace metals. IC_50_ and *K*_i_ values are expressed as mM concentrations.

Species	Tissue	Cd^2+^ (mM)	Cu^2+^	Hg^2+^	Zn^2+^	Co^2+^	Pb^2+^	Ag^+^	Ref.
*Callinectes sapidus*	gills	Ki 0.1 × 10^−3^	Ki 3.6 × 10^−3^		Ki 2–6 × 10^−3^			Ki 0.05 × 10^-3^	[51]
*Carcinus maenas*	gills	Ki 0.6–2.5	Ki 0.6–2.5		Ki 0.6–2.5				[51]
*Anguilla anguilla*	gills	IC_50_ 9.9 × 10^−3^							[46]
*Anguilla anguilla*	intestine	IC_50_ 36.4 × 10^−3^							[46]
*Sparus aurata*	liver	Ki 17.7 (non-competitive)	Ki 36.2 (non-competitive)					Ki 0.02 (non-competitive)	[48]
*Ictalurus punctatus*	erythrocyte	IC_50_ 0.9	IC_50_ 0.065		IC_50_ 0.7			IC_50_ 0.035	[44]
*Oncorhynchus mykiss*	brain	Ki 94.2 × 10^−3^	Ki 27.6 × 10^−3^ (non-competitive)		Ki 1.20	Ki 0.035			[49]
*Acipenser gueldenstaedti*	erythrocyte		IC_50_ 5.2		IC_50_ 2.8			IC_50_ 1.7	[53]
*Dicentrarchus labrax*	liver			Ki 0.76	Ki 0.72 (competitive)	Ki 0.53 (competitive)	Ki 0.24 (competitive)		[47]
*Gallus*	erythrocyte		Ki 2.78 (competitive)	Ki 1.26 (competitive)			Ki 0.97 (competitive)		[55]
*Ovis aries*	kidney	Ki 1.04 (competitive)	Ki 4.70 (competitive)		Ki 0.96 (competitive)				[54]
*Homo sapiens*	Erythrocyte, CA I		Ki 3.22	Ki 3.22 (uncompetitive)		Ki 1.45 (competitive)	Ki 1 (non-competitive)		[52]
*Homo sapiens*	Erythrocyte, CA II		Ki 0.312	Ki 0.312 (uncompetitive)		Ki 1.7 (non-competitive)	Ki 0.056 (competitive)		[52]

The inhibitory effect of metals on CA activity demonstrated in *in vitro* studies has been confirmed by works carried out in *in vivo* exposure conditions. In the mantle of the filter-feeding mussel *Mytilus galloprovincialis*, CA was significantly inhibited by exposure to 1.78 µM Cd^2+^ for 15 days [57]. Considering the role of CA in carbonate salt deposition, the inhibitory effect of Cd^2+^ on mantle CA activity could explain the significant decrease in shell growth previously observed in *M. galloprovincialis* exposed to heavy metals. [58]. In the marine anemone *Aiptasia pallida*, a decrease of CA activity was observed after waterborne exposure to metal mixtures (Cu^2+^, Zn^2+^, Ni^2+^ and Cd^2+^) with a significant CA activity reduction following treatment with 100 μg/L for three days [59]. CA activity in the gills of the freshwater bivalve *Anodonta anatine* was significantly inhibited by the exposure to 0.35 µM Cu^2+^ for 15 days [60]. A CA activity decrease with increasing exposure concentrations of Cu^2+^ was observed in two species of scleractinian corals, such as *Acropora cervicornis* and *Montastraea faveolata*. Significant effects were detected in *A. cervicornis* exposed to 10 and 20 μg/L Cu^2+^ and in M. faveolata exposed to 20 μg/L Cu^2+^ for five weeks [61], respectively. A significant decrease in CA activity was recorded also in the anemones *Condylactis gigantea* and *Stichodactyla helianthus* exposed to Cu^2+^, Ni^2+^, Pb^2+^ and V^2+^ for 48 h [62].

From all of the available studies, a great variability in the sensitivity of CA activity to *in vitro* and *in vivo* metal exposure was observed among species, tissues and metals. This great variability can be explained by the complexity and multifaceted aspects of the binding of metals with CA isoforms, producing a number of different inhibitory responses to different metals in different tissues and species. It is possible to hypothesize that structural differences in CA isoforms could generate different metal-binding affinities and in turn different inhibitory responses.

## 4. Metals and CA Protein Expression

The inhibition exerted by some metals on CA-specific isoforms, as a result of the displacement of the native metal cofactor or the binding to other sites in the CA molecule, did not explain all of the biological effects of metals on CA.

It is known in plants and phytoplankton that biosynthesis of CA may be regulated by some environmental factors, including environmental trace metal concentrations [26,63]. Concerning animals, less information is available on this aspect. Some works indicate CA protein expression to be influenced by Zn^2+^ availability. For example, in humans, patients with CAVI deficiency show stimulation of CAVI synthesis/secretion by Zn^2+^ treatment [64], presumably through metal-induced upregulation of the CAIV gene. In rats, zinc deficiency induced a decrease in CAII protein expression in the submandibular gland [65].

In the fish *Cyprinodon variegatus*, CAII gene expression in gills and intestine was influenced by Cu^2+^ exposure in a concentration-dependent manner, with a two-fold upregulation in the gills and a three-fold upregulation in the intestine following nine days of exposure to 100 mg/L Cu^2+^ [66]. The effect of Cu^2+^ on CA expression appears more complex if we consider that the metal in the same fish is also able to interfere with the regulation of CA expression by osmotic stress, disrupting the osmotic stress induction of the intestinal CAII. These results outline that CA plays a role in the combined effects of copper and osmotic stress on ion homeostasis and show how this enzyme represents a factor linking the physiological responses of the organism to multiple stresses.

In mussels, Caricato *et al.* [67] demonstrated that cadmium exposure (1.785 µM CdCl_2_ for 14 days) enhances CA protein expression in digestive gland, as assessed by Western blotting analysis, CA activity measurement and immunofluorescence analysis in both laboratory and field experiments [67,68]. In basal conditions, digestive gland cells show a high CA specific activity, which can be functional to the well-developed lysosomal compartment of these cells. In fact CA, catalyzing the H^+^ production from metabolic CO_2_, can provide the H^+^ necessary for the lysosome acidification. Therefore, the authors hypothesized that the observed metal-induced CA increased expression in mussel digestive gland is functionally related to the activation of the lysosomal system known to occur in pollutant-exposed organisms [69] and well described in mussel digestive gland [70].

The metal induction of CA expression arouses the question of how to interpret the different opposite effects that some metals can exert on CA, such as inhibition of the activity and upregulation of the gene expression. In mussel digestive gland, the IC_50_ value for Cd^2+^ inhibition of CA activity *in vitro* was in the millimolar range, while CA induction was observed following *in vivo* exposure to 1.785 µM CdCl_2_ for two weeks [67]. In this case, it is possible to argue that the effect of Cd^2+^ on CA is dose-dependent with upregulation of CA expression at low concentrations and inhibition of the CA activity at higher concentrations. Moreover, the type of effect of metals on CA, induction *vs.* inhibition, is tissue specific. In fact, in the case of mussels, the same organisms exposed to 1.785 µM CdCl_2_ for two weeks showed mantle CA significantly inhibited [57], while digestive gland CA significantly increased [67].

Although future work is needed to clarify this intriguing aspect and to deepen the research on the regulation of CA expression by metals, these results enrich the panorama of information about the effects of metals on CA, pointing to the importance of the concentration of exposure, the type of exposure and the metal and tissue specificity on the observed effects.

## 5. CA and Trace Metals: Applicative Insights and Perspectives

As emerged from the above described studies, the relationships between metals and CA are complex and multifaceted, metals being cofactors of CA, but also inhibitors of CA activity and modulators of CA expression. In this field, new applicative perspectives have been recently developed in the biotechnological and environmental fields, allowing translating new advances in basic science on the interaction between CA and metals into practice and novel applications.

### 5.1. CA Metallovariants

The ability of many divalent metal ions, such as Co^2+^, Ni^2+^, Mn^2+^, Cu^2+^, Cd^2+^ and Hg^2+^, to easily bind to the three-histidine moiety within the CA active site in transmetallation experiments generated a number of CA metallovariants.

The interest in CA metallovariants goes beyond the study of the metal-protein interaction in the catalytic site structure. It allows probing the changes certain metals make to the function of the enzyme, including new catalytic reactions. This has been observed particularly in CA enzymes containing Co^2+^ and Mn^2+^ metal centers [71]. The natural function of CA is to catalyze the reversible hydration of CO_2_ to HCO_3_^−^, but it is also known for its ability to catalyze the hydrolysis of esters with moderate enantioselectivity. Replacing zinc with manganese in the active site produced the manganese-substituted carbonic anhydrase (CA[Mn]) with peroxidase activity [72]. In the presence of bicarbonate and hydrogen peroxide, CA[Mn] catalyzed the efficient oxidation of *O*-dianisidine with *K*_cat_/*K*_m_ comparable to that for horseradish peroxidase. CA[Mn] also catalyzed the moderately enantioselective epoxidation of olefins to epoxides. This enantioselectivity of CA[Mn] is similar to that observed in natural heme-based peroxidases, with the advantage that CA[Mn] avoids the formation of aldehyde side products [71].

Therefore, the ability of some transition metals to displace the native Zn^2+^ in the catalytic site discloses a CA catalytic promiscuity that has begun to be recognized as a valuable research and synthesis tool in potential biotechnological applications, leading to improvements in existing catalysts and providing novel synthesis pathways currently not available.

### 5.2. CA-Based Biosensors for Metal Ions

Pollution by trace metals is a world-wide problem, which raises concerns about potential effects on human health and the environment [73]. Mining and smelting, industrial and urban waste, wastewater discharges and shipping activity are the major anthropogenic sources of metals in the aquatic environment. Many sensitive and selective analytical methods for the detection of metal ions at low concentrations in environmental samples have been developed. In this field, great is the interest for simple, rapid and inexpensive alternatives to the classical analytical methods, able to provide a continuous analysis of metal concentration *in situ* and in real time. This interest is expressed by the development of biosensors for trace metal detection. Some of them are fluorescence-based biosensors for the determination of free metals in solution [74,75,76], such as Cu^2+^, Co^2+^, Zn^2+^, Cd^2+^ and Ni^2+^ at concentrations down the picomolar range [76,77]. They utilize the affinity of the apoCA (in general variants of human CAII) for metals [76,78].

Therefore, the high affinity of CA for metals has driven the development of CA-based biosensing that has been shown to be a viable approach for determining certain divalent cations in environmental media, serving also as an archetype for other fluorescence-based biosensors [79].

### 5.3. CA-Based Ecotoxicological Biomarkers and Bioassays for Metal Pollution Assessment

In the last few decades, the use of effect-based methodologies, such as ecotoxicological bioassays and biomarkers, has received growing interest in environmental monitoring and assessment. Ecotoxicity bioassays are widely used for the assessment of environmental media quality, because they provide an integrated measurement of the various complex effects exerted by contaminants present in an environmental sample on the test biological system [80,81]. On the other hand, molecular and cellular biomarkers measured in exposed organism in the field can be helpful for detecting the occurrence of pollutant-induced stress syndrome in living organisms and gaining insight regarding the mechanisms causing the observed effects of chemicals [82,83]. Bioassays and biomarkers provide useful information about the degree of exposure to pollutants and the resulting effects on the organisms. In this regard, the use of bioassay/biomarkers provides early warning information useful for improving the processes of environmental risk assessment [84].

Recently, new perspectives on the potential use of CA as pollution biomarkers in environmental biomonitoring rose from the sensitivity of CA activity and expression to trace metals in several species.

Therefore, CA measurements have been included in multi-biomarker approaches on bioindicator organisms in field studies [85]. For example CA activity inhibition measurement in the bioindicator fish *Pimelodus maculatus* has been included in the multi-marker biomonitoring of three water reservoirs along the Paraiba do Sul River in an industrialized area of Brazil [86]. In corals, CA activity inhibition by metal exposure has been related to inhibition of coral growth through alteration of the calcification process, and it has been suggested as a potential biomarker of exposure to metal pollution [61].

The applicability of digestive gland CA activity and expression in a multimarker approach has been also demonstrated in the bioindicator organism *Mytilus galloprovincialis* [67,87] in two studies addressing the ecotoxicological biomonitoring and assessment of a coastal marine area in Southern Italy.

Branchial CA activity measured in the filter-feeding species *Crassostrea rhizophorae* was shown to be very responsive to coastal contamination in a recent study carried out in three human-impacted Brazilian estuaries [88]. Data were consistent with the usefulness of branchial CA in this species as a supporting biomarker for inexpensive and rapid analysis.

All of the available studies demonstrate the applicability of metal-induced CA alterations as pollution biomarkers. However, the successful use of CA as a biomarker in environmental biomonitoring requires a thorough knowledge of the specific metal-induced CA response in the specific bioindicator species used, because of the high species-specific and tissue-specific effects of metals on CA isoforms.

The *in vitro* inhibition of CA activity by metals has been applied by Lionetto *et al.* [57,89,90] to the development of an *in vitro* bioassay sensitive to the synergic effects of metals in a mixture and useful for the toxicity assessment of environmental aqueous samples [90]. This patented method has been recently applied to the monitoring of harbor sediments [68] and of reclaimed waste waters [91], showing a high agreement with standardized *in vivo* bioassays.

**Figure 3:**
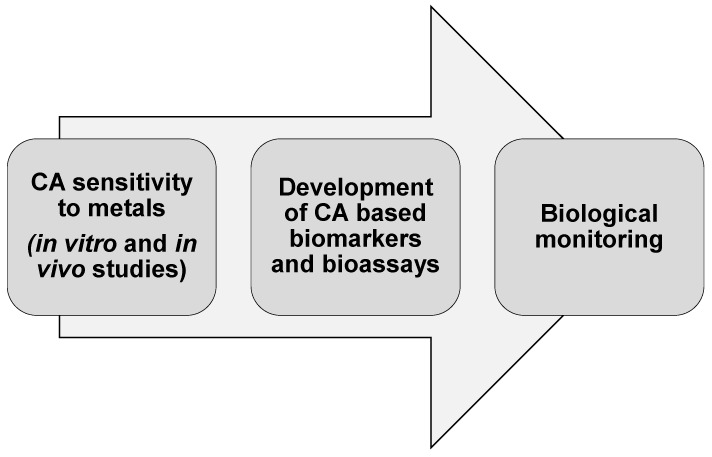
From *in vivo* and *in vivo* assessment of the sensitivity of CA to trace metals to environmental monitoring.

All of the studies carried out to date on the *in vitro* and *in vivo* sensitivity of CA to trace metals contribute to developing effect-based methodologies, such as CA-based pollution biomarkers and bioassays. They allow translating new advances in basic science into practice and novel applications in environmental biomonitoring (Figure 3).

## 6. Conclusions

Although CA has been widely investigated in several aspects of its structure and functions, its complex relationships with metals have been only partially investigated to date, and some questions still remain to be clarified, such as, for example, the metal regulation of CA expression and its underlying mechanisms. This is an intriguing aspect of the research on this metalloenzyme that could raise new perspectives in the understanding of CA function and regulation.

The responses of CA activity and expression to metal exposure in animals need to be further characterized, and this could improve the potentiality of this enzyme for biotechnological and environmental applications.

## References

[1] Supuran C.T. (2010). Carbonic anhydrase inhibitors. Bioorg. Med. Chem. Lett..

[2] Del Prete S., Vullo D., Fisher G.M., Andrews K.T., Poulsen S.A., Capasso C., Supuran C.T. (2014). Discovery of a new family of carbonic anhydrases in the malaria pathogen *Plasmodium falciparum*—The η-carbonic anhydrases. Bioorg. Med. Chem. Lett..

[3] Ivanov B.N., Ignatova L.K., Romanova A.K. (2007). Diversity in forms and functions of carbonic anhydrase in terrestrial higher plants. Russ. J. Plant Physiol..

[4] Cannon G.C., Heinhorst S., Kerfeld C.A. (2010). Carboxysomal carbonic anhydrases: Structure and role in microbial CO_2_ fixation. BBA Proteins Proteom..

[5] Neri D., Supuran C.T. (2011). Interfering with pH regulation in tumors as a therapeutic strategy. Nat. Rev. Drug Discov..

[6] Supuran C.T. (2008). Carbonic anhydrases: Novel therapeutic applications for inhibitors and activators. Nat. Rev. Drug Discov..

[7] Kimber M.S., Pai E.F. (2000). The active site architecture of *Pisum sativum* β-carbonic anhydrase is a mirror image of that of α-carbonic anhydrases. EMBO J..

[8] Smith K.S., Ferry J.G. (2000). Prokaryotic carbonic anhydrases. FEMS Microbiol. Rev..

[9] Alber B.E., Ferry J.G. (1994). A carbonic anhydrase from the archeon *Methanosarcina thermophile*. Proc. Natl. Acad. Sci. USA.

[10] Roberts S.B., Lane T.W., Morel F.M.M. (1997). Carbonic anhydrase in the marine diatom *Thalassiosira weissflogii* (Bacillariophyceae). J. Phycol..

[11] Tripp B.C., Smith K.S., Ferry J.G. (2001). Carbonic anhydrase: New insights for an ancient enzyme. J. Biol. Chem..

[12] Xu Y., Supuran C.T., Morel F.M.M. (2011). Cadmium-Carbonic Anhydrase. Encyclopedia of Inorganic and Bioinorganic Chemistry.

[13] Supuran C.T., Scozzafava A., Casini A. (2003). Carbonic anhydrase inhibitors. Med. Res. Rev..

[14] Supuran C.T., Scozzafava A. (2007). Carbonic anhydrases as targets for medicinal chemistry. Bioorg. Med. Chem. Lett..

[15] Innocenti A., Scozzafava S., Parkkila L., Puccetti G., de Simone G., Supuran C.T. (2008). Investigations of the esterase, phosphatase, and sulfatase activities of the cytosolic mammalian carbonic anhydrase isoforms I, II, and XIII with 4-nitrophenyl esters as substrates. Bioorg. Med. Chem. Lett..

[16] De Simone G., di Fiore A., Capasso C., Supuran C.T. (2015). The zinc coordination pattern in the eta-carbonic anhydrase from *Plasmodium falciparum* is different from all other carbonic anhydrase genetic families. Bioorg. Med. Chem. Lett..

[17] Berg J.M., Tymoczko J.L., Stryer L. (2012). Biochemistry.

[18] Williams R.J.P. (1987). The biochemistry of zinc. Polyhedron.

[19] Maret W. (2013). Zinc biochemistry: From a single zinc enzyme to a key element of life. Adv. Nutr..

[20] Vahrenkamp H. (2007). Why does nature use zinc—A personal view. Dalton Trans..

[21] Supuran C.T. (2012). Structure-based drug discovery of carbonic anhydrase inhibitors. J. Enzym. Inhib. Med. Chem..

[22] MacAuley S.R., Zimmerman S.A., Apolinario E.E., Evilia C., Hou Y., Ferry J.G., Sowers K.R. (2009). The archetype γ-class carbonic anhydrase (Cam) contains iron when synthesized *in vivo*. Biochemistry.

[23] Tripp B.C., Bell C.B., Cruz F., Krebs C., Ferry J.G. (2004). A role for iron in an ancient carbonic anhydrase. J. Biol. Chem..

[24] Ferry J.G. (2010). The gamma class of carbonic anhydrases. Biochim. Biophys. Acta.

[25] Wu Y., Zhao X., Li P., Huang H. (2007). Impact of Zn, Cu, and Fe on the Activity of Carbonic Anhydrase of Erythrocytes in Ducks. Biol. Trace Elem. Res..

[26] Lane T.W., Morel F.M.M. (2000). A biological function for cadmium in marine diatoms. Proc. Natl. Acad. Sci. USA.

[27] Lane T.W., Saito M.A., George G.N., Pickering I.J., Prince R.C., Morel F.M.M. (2005). Biochemistry: A cadmium enzyme from a marine diatom. Nature.

[28] Park H., McGinn P.J., Morel F.M.M. (2008). Expression of cadmium carbonic anhydrase of diatoms in seawater. Aquat. Microbial. Ecol..

[29] Lionetto M.G., Lionetto M.G., Vilella S., Trischitta F., Cappello M.S., Giordano M.E., Schettino T. (1998). Effects of CdCl_2_ on electrophysiological parameters in the intestine of the teleost fish, *Anguilla anguilla*. Aquat. Toxicol..

[30] Yee D., Morel F.M.M. (1996). *In vivo* substitution of zinc by cobalt in carbonic anhydrase of a marine diatom. Limnol. Oceanogr..

[31] Mahon B.P., Pinar M.A., McKenna R. (2015). Targeting Carbonic Anhydrase IX Activity and Expression. Molecules.

[32] Dutta S., Goodsell D. Carbonic anhydrase. http://pdb101.rcsb.org/motm/49.

[33] Håkansson K., Wehnert A., Liljas A. (1994). X-ray analysis of metal-substituted human carbonic anhydrase II derivatives. Acta Crystallogr. D.

[34] Kogut K.A., Rowlett R.S. (1987). A comparison of the mechanisms of CO_2_ hydration by native and Co^2+^-substituted carbonic anhydrase II. J. Biol. Chem..

[35] Marino T., Russo N., Toscano M. (2005). A Comparative Study of the Catalytic Mechanisms of the Zinc and Cadmium Containing Carbonic Anhydrase. J. Am. Chem. Soc..

[36] Led J.J., Neesgaard E. (1987). Paramagnetic Carbon-13 NMR Studies of the Kinetics and Mechanism of the HCO_3_/CO_2_ Exchange Catalyzed by Manganese(II) Human Carbonic Anhydrase I. Biochemistry.

[37] Sven L. (1997). Structure and mechanism of carbonic anhydrase. Pharmacol. Ther..

[38] Hoffmann K.M., Samardzic D., van den Heever K., Rowlett R.S. (2011). Co(II)-substituted *Haemophilus influenzae* b-carbonic anhydrase: Spectral evidence for allosteric regulation by pH and bicarbonate ion. Arch. Biochem. Biophys..

[39] Eriksson E.A., Kylsten P.M., Jones A.T., Liljas A. (1988). Crystallographic studies of inhibitor binding sites in human carbonic anhydrase II; A penta coordinated binding of the SCN- ion to the zinc at high pH. Proteins.

[40] Hogeback J., Schwarzer M., Wehe C.A., Sperlingab M., Karst U. (2015). Investigating the adduct formation of organic mercury species with carbonic anhydrase and hemoglobin from human red blood cell hemolysate by means of LC/ESI-TOF-MS and LC/ICP-MS. Metallomics.

[41] Tu C., Wynns G.C., Silverman D.N. (1981). Inhibition by cupric ions of ^18^O exchange catalyzed by human carbonic anhydrase II. Relation to the interaction between carbonic anhydrase and hemoglobin. J. Biol. Chem..

[42] Song H., Weitz A.C., Hendrich M.P., Lewis E.A., Emerson J.P. (2013). Building reactive copper centers in human carbonic anhydrase II. J. Biol. Inorg. Chem..

[43] Nettles W.L., Song H., Farquhar E.R., Fitzkee N.C., Emerson J.P. (2015). Characterization of the copper(II) binding sites in human carbonic anhydrase II. Inorg. Chem..

[44] Christensen G.M., Tucker J.H. (1976). Effects of selected water toxicants on the *in vitro* activity of fish carbonic anhydrase. Chem. Biol. Interact..

[45] Lionetto M.G., Maffia M., Cappello M.S., Giordano M.E., Storelli C., Schettino T. (1998). Effect of cadmium on carbonic anhydrase and Na^+^-K^+^-ATPase in eel, *Anguilla anguilla*, intestine and gills. Comp. Biochem. Physiol. A.

[46] Lionetto M.G., Giordano M.E., Vilella S., Schettino T. (2000). Inhibition of eel enzymatic activities by cadmium. Aquat. Toxicol..

[47] Ceyhun S.B., Şentürk M., Yerlikaya E., Erdoğan O., Küfrevioğlu Ö.I., Ekinci D. (2011). Purification and characterization of carbonic anhydrase from the teleost fish *Dicentrarchus labrax* (European Seabass) liver and toxicological effects of metals on enzyme activity. Environ. Toxicol. Pharmacol..

[48] Kaya E.D., Söyüt H., Beydemir S. (2013). Carbonic anhydrase activity from the gilthead sea bream (*Sparus aurata*) liver: The toxicological effects of heavy metals. Environ. Toxicol. Pharmacol..

[49] Soyut H., Beydemir Ş., Hisar O. (2008). Effects of some metals on carbonic anhydrase from brains of rainbow trout. Biol. Trace Elem. Res..

[50] Vitale A.M., Monserrat J.M., Casthilo P., Rodriguez E.M. (1999). Inhibitory effects of cadmium on carbonic anhydrase activity and ionic regulation of the estuarine crab, *Chasmagnathus granulata* (Decapoda, Grapsidae). Comp. Biochem. Physiol. C.

[51] Skaggs H.S., Henry R.P. (2002). Inhibition of carbonic anhydrase in the gills of two euryhaline crabs, *Callinectes sapidus* and *Carcinus maenas*, by heavy metals. Comp. Biochem. Physiol. C.

[52] Ekinci D., Beydemir Ş., Küfrevioğlu Ö.İ. (2007). *In vitro* inhibitory effects of some heavy metals on human erythrocyte carbonic anhydrases. J. Enzym. Inhib. Med. Chem..

[53] Kolayli S., Karahalil F., Sahin H., Dicer B., Supuran C.T. (2011). Characterization and inhibition studies of an α-carbonic anhydrase from the endangered sturgeon species *Acipenser gueldenstaedti*. J. Enzym. Inhib. Med. Chem..

[54] Demirdağ R., Comakli V., Kuzu M., Yerlikaya E., Şentürk M. (2013). Purification and characterization of carbonic anhydrase from Ağrı Balık Lake Trout Gill (*Salmo trutta* labrax) and effects of sulfonamides on enzyme activity. J. Biochem. Mol. Toxicol..

[55] Mercan L., Ekinci D., Supuran C.T. (2014). Characterization of carbonic anhydrase from Turkish native “Gerze” chicken and influences of metal ions on enzyme activity. J. Enzym. Inhib. Med. Chem..

[56] Jebali J., Chouba L., Bannia M., Boussetta H. (2014). Comparative study of the bioaccumulation and elimination of trace metals (Cd, Pb, Zn, Mn and Fe) in the digestive gland, gills and muscle of bivalve *Pinna nobilis* during a field transplant experiment. J. Trace Elem. Med. Biol..

[57] Lionetto M.G., Caricato R., Erroi E., Giordano M.E., Schettino T. (2006). Potential application of carbonic anhydrase activity in bioassay and biomarker studies. Chem. Ecol..

[58] Soto M., Ireland M.P., Marigómez I. (2000). Changes in mussel biometry on exposure to metals: Implications in estimation of metal bioavailability in “Mussel-Watch” programmes. Sci. Total Environ..

[59] Brock J.R., Bielmyer G.K. (2013). Metal accumulation and sublethal effects in the sea anemone, *Aiptasia pallida*, after waterborne exposure to metal mixtures. Comp. Biochem. Physiol. C.

[60] Santini O., Chahbane N., Vasseur P., Frank H. (2011). Effects of low-level copper exposure on Ca^2+^-ATPase and carbonic anhydrase in the freshwater bivalve *Anodonta anatine*. Toxicol. Environ. Chem..

[61] Bielmyer G.K., Grosell M., Bhagooli R., Baker A.C., Langdon C., Gillette P., Capo T.R. (2010). Differential effects of copper on three species of scleractinian corals and their algal symbionts (*Symbiodinium* spp.). Aquat. Toxicol..

[62] Gilbert A.L., Guzman H.M. (2001). Bioindication potential of carbonic anhydrase activity in anemones and corals. Mar. Pollut. Bull..

[63] Khan N.A., Singh S., Anjum N.A., Nazar R. (2008). Cadmium effects on carbonic anhydrase, photosynthesis, dry mass and antioxidative enzymes in wheat (*Triticum aestivum*) under lowand sufficient zinc. J. Plant Interact..

[64] Henkin R., Martin B.M., Agarwal R. (1999). Efficacy of exogenous oral zinc in treatment of patients with carbonic anhydrase VI deficiency. Am. J. Med. Sci..

[65] Goto T., Shirakawa H., Furukawa Y., Komai M. (2008). Decreased expression of carbonic anhydrase isozyme II, rather than of isozyme VI, in submandibular glands in long term zinc-deficient rats. Br. J. Nutr..

[66] De Polo A., Margiotta-Casaluci L., Lockyer1 A.E., Scrimshaw M.D. (2014). A new role for carbonic anhydrase 2 in the response of fish to copper and osmotic stress: Implications for multi-stressor studies. PLoS ONE.

[67] Caricato R., Lionetto M.G., Dondero F., Viarengo A., Schettino T. (2010). Carbonic anhydrase activity in *Mytilus galloprovincialis* digestive gland: Sensitivity to heavy metal exposure. Comp. Biochem. Physiol. C.

[68] Lionetto M.G., Caricato R., Giordano M.E., Erroi E., Schettino T. Carbonic anydrase and chemical pollutants: New applied perspectives. Proceedings of the 65th Annual Meeting of the Italian Physiological Society.

[69] Köhler A. (1991). Lysosomal perturbations in fish liver as indicators for toxic effects of environmental pollution. Comp. Biochem. Physiol..

[70] Moore M.N., Simpson M.G. (1992). Molecular and cellular pathology in environmental impact assessment. Aquat. Toxicol..

[71] Jing Q., Okrasa K., Kazlauskas R.J. (2009). Manganese-Substituted α-Carbonic Anhydrase as an Enantioselective Peroxidase. Top. Organomet. Chem..

[72] Okrasa K., Kazlauskas R.J. (2006). Manganese-Substituted Carbonic Anhydrase as a New Peroxidase. Chem. Eur. J..

[73] De Mora S., Fowler S.W., Wyse E., Azemard S. (2004). Distribution of heavy metals in marine bivalves, fish and coastal sediments in the Gulf and Gulf of Oman. Mar. Pollut. Bull..

[74] Zeng H.H., Thompson R.B., Maliwal B.P., Fones G.R., Moffett J.W., Fierke C.A. (2003). Real-time determination of picomolar free Cu(II) in seawater using a fluorescence-based fiber optic biosensor. Anal. Chem..

[75] Bozym R., Hurst T.K., Westerberg N., Stoddard A., Fierke C.A., Frederickson C.J., Thompson R.B. (2008). Determination of zinc using carbonic anhydrase-based fluorescence biosensors. Methods Enzymol..

[76] Fierke C.A., Thompson R.B. (2001). Fluorescence-based biosensing of zinc using carbonic anhydrase. Biometals.

[77] Mei Y.J., Frederickson C.J., Giblin L.J., Weiss J.H., Medvedeva Y., Bentley P.A. (2011). Sensitive and selective detection of zinc ions in neuronal vesicles using PYDPY1, a simple turn-on dipyrrin. Chem. Commun..

[78] McCall K.A., Fierke C.A. (2004). Probing determinants of the metal ion selectivity in carbonic anhydrase using mutagenesis. Biochemistry.

[79] Thompson R.B., Bozym R.A., Cramer M.L., Stoddard A.K., Westerberg N.M., Fierke C.A., Thompson R.B. (2005). Chapter 6: Carbonic anhydrase-based biosensing of metal ions: Issue and future prospects. Fluorescence Sensors and Biosensors.

[80] Costa C.R., Olivi P., Botta C.M.R., Espindola E.L.G. (2008). Toxicity in aquatic environments: Discussion and evaluation methods. Quim Nova.

[81] Manzo S., de Nicola F., de Luca Picione F., Maisto G., Alfani A. (2008). Assessment of the effects of soil PAH accumulation by a battery of ecotoxicological tests. Chemosphere.

[82] Forbes V.E., Palmqvist A., Bach L. (2006). The use and misuse of biomarkers in ecotoxicology. Environ. Toxicol. Chem..

[83] Schettino T., Caricato R., Calisi A., Giordano M.E., Lionetto M.G. (2012). Biomarker approach in marine monitoring and assessment: New insights and perspectives. Open Environ. Sci..

[84] Martίnez-Gόmez C., Vethaak A.D., Hylland K., Burgeot T., Khöler A., Lyons B.P., Thain J., Gubbins M.J., Davies I.M. (2010). A guide to toxicity assessment and monitoring effects at lower levels of biological organization following marine oil spills in European waters. J. Mar. Sci..

[85] Lionetto M.G., Caricato R., Giordano M.E., Erroi E., Schettino T. (2012). Carbonic anhydrase as pollution biomarker: An ancient enzyme with a new use. Int. J. Environ. Res. Public Health.

[86] De Andrade Brito I., Freire C.A., Yamamoto F.Y., de Assis H.C.S., Souza-Bastos L.R., Cestari M.M., de Castilhos Ghisi N., Prodocimo V., Filipak Neto F., de Oliveira Ribeiro C.A. (2012). Monitoring water quality in reservoirs for human supply through multi-biomarker evaluation in tropical fish. J. Environ. Monit..

[87] Caricato R., Lionetto M.G., Schettino T. (2009). Studio di biomarkers in mitili (*Mytilus galloprovincialis*) traslocati in Mar Piccolo e in Mar Grande di Taranto. Biol. Mar. Medit..

[88] Azevedo-Linhares M., Freire C.A. (2015). Evaluation of impacted Brazilian estuaries using the native oyster *Crassostrea rhizophorae*: Branchial carbonic anhydrase as a biomarker. Ecotoxicol. Environ. Saf..

[89] Lionetto M.G., Caricato R., Erroi E., Giordano M.E., Schettino T. (2005). Carbonic anhydrase based environmental bioassay. Int. J. Environ. Anal. Chem..

[90] Schettino T., Lionetto M.G., Erroi E. (2012). Method for enzymatic assessment of the toxicity of environmental aqueous matrices.

[91] Lionetto M.G., Caricato R., Calisi A., Giordano M.E., Erroi E., Schettino T. (2015). Biomonitoring of water and soil quality: A case study of ecotoxicological methodology application to the assessment of reclaimed agroindustrial wastewaters used for irrigation. Rend. Fis. Acc. Lincei.

